# Comparative analysis of root associated microbes in tropical cultivated and weedy rice (*Oryza* spp.) and temperate cultivated rice

**DOI:** 10.1038/s41598-024-60384-0

**Published:** 2024-04-26

**Authors:** Vani Juliyanti, Ryota Itakura, Kanta Kotani, Shu Yong Lim, Go Suzuki, Chun Wie Chong, Beng Kah Song, Sadequr Rahman

**Affiliations:** 1 School of Science, Monash University Malaysia, Bandar Sunway, Malaysia; 2 Division of Natural Science, Osaka Kyoiku University, Kashiwara, 582-8582 Japan; 3 Graduate School of Biostudies, Kyoto University, Sakyo-ku, Kyoto, 606-8501 Japan; 4 Genomics Facility, Monash University Malaysia, Bandar Sunway, Malaysia; 5 School of Pharmacy, Monash University Malaysia, Bandar Sunway, Malaysia; 6 Tropical Medicine and Biology Multidisciplinary Platform, Monash University Malaysia, Bandar Sunway, Malaysia

**Keywords:** Microbiology, Molecular biology

## Abstract

Weedy rice is a major problem in paddy fields around the world. It is well known that weedy rice appears to grow faster and mature earlier than cultivated rice. It is possible that differences in the root microbial genetics are correlated with this characteristic. This study incorporated 16S rRNA amplicon sequencing to study the microbial composition in the rhizosphere and endosphere of rice root. No significant difference was found between the microbiota associated with weedy and cultivated rice lines grown in the same field. It was found that the endosphere had less microbial diversity compared to the rhizosphere. The major groups of bacteria found in the endosphere are from the phylum Proteobacteria, Myxococcota, Chloroflexota, and Actinobacteria. In addition, by analyzing the microbiome of *japonica* rice grown in the field in a temperate climate, we found that despite differences in genotype and location, some bacterial taxa were found to be common and these members of the putative rice core microbiome can also be detected by in situ hybridization. The delineation of a core microbiome in the endosphere of rice suggests that these bacterial taxa might be important in the life cycle of a wide range of rice types.

## Introduction

Rice is one of the most important cereal crops around the world and is the main source of carbohydrates for many people globally. Global rice production reached 520.8 million tonnes in 2021 on a milled basis^1^. There is a long history of rice domestication involving natural and artificial selection. Indeed, more than 120,000 rice varieties are cultivated worldwide^2^ with different agronomic and nutritional traits^3,4^.

The productivity of a rice field depends on the surrounding biotic and abiotic factors. Among the biotic factors, the genotype of the cultivated rice plant and the degree of weed infestation in the field play an important role. As a conspecific relative of domesticated rice (*Oryza sativa*), weedy rice (*Oryza* spp.) poses a significant threat to the rice industry worldwide, owing to its wild-like phenotypic characteristics such as easy seed shattering, seed dormancy and better nutrient uptake^5–8^. Depending on the degree of the infestation, a weedy rice outbreak can cause a reduction in yield production by up to 80%^9^. Generally, compared to domesticated rice, weedy rice has higher efficiency in the uptake and usage of nutrients^8^. The rapid growth of weedy rice suggests that it is more efficient in acquiring nutrients from the environment than cultivated rice.

Root and soil microbe interaction is an essential biotic factor for rice productivity^10^. The close association between roots and microorganisms may indicate adaptive cooperation, in which the microbes convert nutrients in the soil into usable form for plants. In return, the root exudates, which contain carbon metabolites, can serve as a useful energy source for the growth of microorganisms^11^. The close association between plants and soil microbes confers a survival advantage for plants against environmental perturbation^12–14^.

Weedy rice generally grows faster and matures earlier than cultivated rice^5^. However, the reasons are unknown. We speculated that the faster growth rate observed in weedy rice might be attributed to the difference in the root microbial composition. Rice microbiomes have been described for many cultivated rice lines, for instance, rice cultivated in pots in the USA, in well controlled environments in China and in the field in Ghana, Africa^15–17^. However, the microbiome from root endosphere of a tropical rice line and its closely related weedy rice counterpart have not been described and compared.

There are three main compartments of rice root systems including rhizosphere, rhizoplane and endosphere. The rhizosphere refers to the soil surrounding roots and this is affected by plant root exudates, the rhizoplane is related to the outer root surface and the endosphere is the area inside the root system. Each compartment of the root harbours different types of microbial communities^15^. The microbiota associated with the rhizosphere in *indica* and *japonica* rice have been described using high throughput 16S sequencing but the microbiota in the endosphere and localization in different root components were not compared^18^.

In this study, we investigate microbial diversity in the endosphere and rhizosphere of an *indica* rice grown in a tropical paddy field using high throughput amplicon sequencing. The microbiome of the endosphere and rhizosphere of coexisting weedy rice in the same environment was also analyzed. In addition, we describe and compare the microbiome of a *japonica* rice line grown in Japan with the *indica* cultivated rice in Malaysia and show that despite the differences, common components of the microbiota can be surmised, both by sequencing and by fluorescent in situ hybridization.

## Materials and methods

### Plant materials for microbiome analysis

#### *Indica* rice samples

A total of 108 samples comprising nine pairs each of cultivated (*Oryza sativa* cv*. MR269, MR297, MR253*) and weedy rice roots and rhizosphere soil representing three sampling sites across two states of Peninsular Malaysia, were collected in 2019. Three pairs of cultivated and weedy rice samples were collected from each sampled location. Two of the locations occur in Kelantan state (Ketereh: 5° 56′ 49.416″ N, 102° 15′ 49.781″ E and Machang: 5° 45′ 25.056″ N, 102° 12′ 16.662″ E) and one in Selangor state (Sekinchan: 3° 31′ 45.84″ N, 101° 7′ 26.399″ E). Each root sample was divided into three biological replicates and stored in the cold phosphate buffer saline (PBS). All samples were kept at − 80 °C until further processing.

#### *Japonica *rice ﻿samples

A total of sixteen rice root samples from four cultivated rice plants (*Oryza sativa* cv. *Hinohikari*) grown on the paddy field in Kyoto, Japan (35° 03′ 11″ N, 135° 47′ 16″ E) were collected before heading in 2021. All samples were kept at − 80 °C.

### Separation of rhizosphere from endosphere by using sonication method for *indica* rice group

The eighteen samples collected were processed by taking three biological replicates per sample and separating the rhizosphere and endosphere fractions, making it a total of 108 samples (Supplementary Data S1). The excess soil was removed by manual shaking of the root. The soil tightly attached to the root was left to be separated as rhizosphere fraction. Each root sample was cut into 0.25–0.30 g and placed into a falcon tube with 1 × PBS. Each tube was vortexed to separate the rhizospheric soil from the root surface. The process was repeated twice to ensure that most of the rhizospheric soil is removed.

The sonication procedures were adapted from the study by Lundberg et al.^19^ with minor adjustments. Briefly, the PBS containing rhizosphere fraction was passed through the 100 µL cell strainer to further separate the soil from any possible rock and other plant tissue debris which were still left in the buffer to obtain a fine rhizosphere fraction. After that, the fine soil in each tube was centrifugated at 3200 *g* for 15 min at 10 °C and most of the supernatant was removed. The concentrated soil was removed into microcentrifuge tubes. The soil in the microcentrifuge tube was centrifugated again at 10,000 *g* for 5 min and the supernatant was removed. The pellet was stored at − 80 °C.

The root tissue in clean 1 × PBS was sonicated in the ultrasonic bath sonicator (power = 5, 30 s burst, 30 s rest, for 5 cycles). The PBS was changed and followed by the second round of sonication. The same applied to the third round of sonication. After finishing the three rounds of sonication, the root tissues were moved into a new falcon tube with fresh PBS and shaken lightly for rinsing. After that, the root tissues were stored with fresh PBS and kept at − 80 °C.

### DNA extraction from rhizosphere and endosphere

The cleaned root tissue was first homogenized in the FastPrep 24™ 5G (MP Biomedicals, Irvine, CA, USA) (speed set to 6, time was 40 s × 2). DNeasy PowerSoil DNA isolation kit (Qiagen, Valencia, CA, USA) was used to extract DNA from the rhizosphere and roots (endosphere).

The quality and quantity of the extracted DNA samples were checked using Qubit® 3.0 Fluorometer (Life Technologies, Carlsbad, CA, USA). Only the samples with concentration of at least 1 ng/µL.

### Generation of amplicon, library preparation and sequencing

PCR amplification was carried out on the extracted DNA for each sample by incorporating the 341F/805R primer pair to target V3–V4 hypervariable region of bacterial 16S rRNA gene using standard conditions, including denaturation at 95 °C for 3 min, followed by 25 cycles of denaturation, annealing and extension at 95 °C, 53 °C and 72 °C respectively and final extension was at 72 °C for 1 min.

Library preparation was performed by using Nextera DNA library prep kit (Illumina, San Diego, CA, USA) followed by quality checking of the generated library using Qubit® 2.0 Fluorometer (Life Technologies) and Agilent 2200 TapeStation (Agilent Technologies, CA, USA). Sequencing was conducted using an Illumina MiSeq platform at Genomics Facility, Monash University, Malaysia.

### DNA isolation of *japonica* rice samples from Japan

These were prepared by a slightly different method. The rhizosphere was not collected and the endosphere was obtained by the following:

Rice roots were rinsed with running water for 5 min, and further washed by vortex treatment for 30 s in 1 × PBS. The roots were sonicated in 1 × PBS for 30 s using an ultrasonic homogenizer (VP-050, TAITEC) 3 times. Sonicated roots were rinsed with PBS and used for bacterial DNA isolation using DNeasy Plant Mini Kit (QIAGEN, Valencia, CA, USA) with slight modification by adding Proteinase K treatment.

Biological replicates of *indica* samples were also tested with these DNA extraction protocols and the results showed that this protocol produced results that clustered together with the above *indica* protocol. Hence, the *japonica* samples can be included in the analysis (Supplementary Figure 7).

### FISH analysis

Rice plants (*O. sativa* cv. *Hinohikari*) grown in pots in Osaka Kyoiku University and the paddy field in Hyogo, Japan were collected before heading. For fixation, the roots were rinsed with running water for 5 min. For plastic resin embedding, rice root tissues were transferred into 1 × PBS, washed with a vortex mixer for 30 s, and fixed in 4% paraformaldehyde (PFA) with 1 × PBS overnight at 4 °C, followed by storage in 50% ethanol with PBS at − 20 °C. After fixation, the tissue samples were dehydrated by using the 50, 70, 80, 90, 95, and 100% ethanol, then embedded in Technovit 7100 (Kulzer), and cut into 10 or 15 µm-thickness for sections by using a rotary microtome (RV-240, Yamato Kohki Industrial). For specimen preparation, the sections were spread on the MAS-coated slide glass (S9116, Matsunami Glass).

As for the preparation of FISH probes, the nucleotide sequence of 16S rRNA-specific oligonucleotide probes was searched using the probeBase (http://probebase.csb.univie.ac.at/) website. The probes of SRB385Db (5′-CGG CGT TGC TGC GTC AGG-3′) for Myxococcales and DEN441 (5′-TGC GAT TTC TTC CCG GCC-3′) for Rhodocyclaceae were labelled with FITC. The EUB338 probe (5′-GCT GCC TCC CGT AGG AGT-3′) for detecting most bacteria was labelled with Cy3.

For FISH experiments, after permeabilization by incubation with 2.0 mg/mL lysozyme at room temperature for 30 min, the samples were washed three times with 1 × PBS chilled on ice and dried with a blower. The 10 µL solution including the 1 pmol/µL FISH probes with hybridization buffer (0.9 M NaCl, 0.02 M Tris HCl pH 7.2, 0.01% SDS, 10% formamide) was dropped on the section specimen, and overnight hybridization was performed at 37 °C. The samples were rinsed by washing buffer I (0.9 M NaCl, 0.02 M Tris HCl pH 7.2, 0.11% SDS) at room temperature, and were incubated at 37 °C for 20 min in washing buffer I and then incubated at 37 °C for 15 min in washing buffer II (0.18 M NaCl, 0.02 M Tris HCl pH 7.2, 0.01% SDS). After additional rinsing with sterilized water, the slides were dried with a blower, and mounted in a fluorescence antifade solution (DABCO) and a counter staining DAPI solution. The FITC and Cy3 signals on the slides were captured with an Axioskop fluorescence microscope (Zeiss) coupled to a cooled CCD camera (model 4880, Hamamatsu Photonics).

### Bioinformatics analysis for 16S rRNA

Both forward and reverse primers were removed from all sequences using Cutadapt v2.10^20^. Additional trimming for adapters was performed by using Fastp v0.20.1^21^. All pre-processed 16S sequences were imported as QIIME 2 *artifacts* file and analyzed in Qiime2 v2022.2^22^. The denoising procedure was carried out with DADA2 to detect and correct Illumina amplicon sequence data including filtering, dereplication, identification of chimera, sample inference and merging the paired end sequences^23^.

A total of 18,449,230 paired-end-reads for all samples were obtained after filtering the low-quality sequences. The median read count was 117,209.5, the mean number of reads was 148,784.1 and the minimum read count was 98,279. The rarefaction curve presenting the relationship of diversity and number of reads showed a stable plateau indicating sufficient number of reads for further analysis (Supplementary Figure 6).

Filtering to remove chloroplast and mitochondria sequences and singleton were performed. Processed reads were obtained and classified into 6,385 amplicon sequence variants (ASVs). The taxonomy of the ASVs was classified by referencing the GreenGenes2 2022.10 database. The taxonomy and abundance table were used for alpha and beta diversity analyses and imported into the R program for visualization. Furthermore, taxonomy analysis based on GreenGenes2 (http://ftp.microbio.me/greengenes_release/2022.10/) database was obtained and a taxa bar plot was generated.

Alpha diversity analysis was performed to investigate the richness and the evenness of microbial communities in the rhizosphere and endosphere of each rice plant. The alpha diversity was inferred using indices such as Faith phylogenetic diversity (Faith_pd), Shannon diversity index, Chao1 index and Pielou’s evenness index. Statistical significance was determined using Kruskal–Wallis Test.

Faith phylogenetic diversity, which is based on the sum of branch lengths from the phylogenetic tree, was used to estimate the within sample diversity. Shannon diversity is based on Claude Shannon’s formula that allows us to measure the richness and evenness of the species. Chao1 is a non-parametric estimator for the richness in the sample, while Pielou’s evenness measures the abundance level of one species to another in the sample group.

Beta diversity was calculated using Aitchison Distance. The distribution of the samples was visualized using principal coordinate analysis (PCoA), while the significance of compositional difference was measured using permutational multivariate analysis of variance (PERMANOVA). Predictive functional profiling was performed with PICRUSt2^24^. The putative metabolic pathway was further examined on MetaCyc database^25^. Separately, differentially abundant bacterial taxa and functional features were identified using the ANalysis of Composition of Microbiomes (ANCOM)^26^.

### Ethics declarations

Experimental research and field studies on plants (either cultivated or wild), including the collection of plant materials complied with relevant institutional, national, and international guidelines and legislation.

## Results and discussion

### Microbial richness and evenness across different root compartments, rice fields and rice types

**Figure 1:**
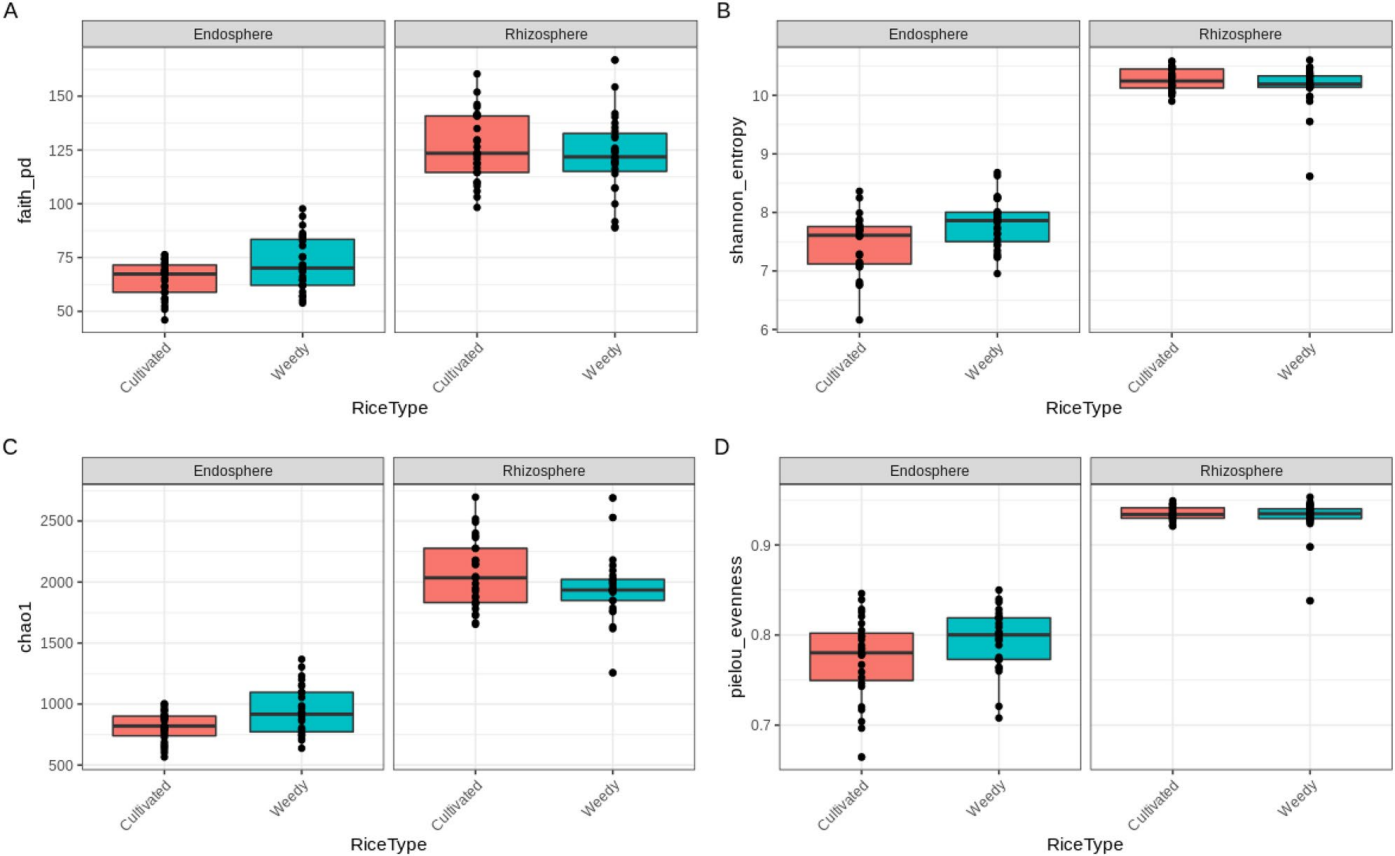
(**a**) Faith’s phylogenetic diversity, (**b**) Shannon diversity, (**c**) Chao1 index indicating bacteria community richness and (**d**) Pielou’s evenness index indicating community evenness in the endosphere and rhizosphere of cultivated and weedy rice.

The community richness and evenness of rhizosphere and endosphere respectively were not significantly different (*p* > 0.05) between cultivated and weedy rice (Fig. 1). However, rhizosphere harboured higher diversity and evenness than endosphere (*p* < 0.01) (Fig. 1). This agrees with reported decrease of microbial richness from rhizosphere to endosphere^15,27^.

The rhizosphere has dynamic and diverse microbial communities which are primarily affected by various biotic and abiotic factors such as the plant genotype, root exudates, soil type, agricultural practices and complex plant-microbes interaction in the fields^28–31^. Despite the selective assembly of microbes in the rhizosphere, complex interaction between microbes and root allows further selection of microbes to the endosphere^32^. In the present study, we identified 22 bacteria taxa uniquely located within the endosphere (Supplementary Figure 4, Supplementary Data S4). These could be produced through selective entry into the plant roots or perhaps derived from the inherent bacteria populations of the seeds, transmitted vertically to the next generation of the plants^33^. It has also been found that endogenous microbes in the seedlings of rice plants can be transmitted to the roots and stems throughout plant development^16^.

### Investigation of microbial diversity in the endosphere and rhizosphere

**Figure 2:**
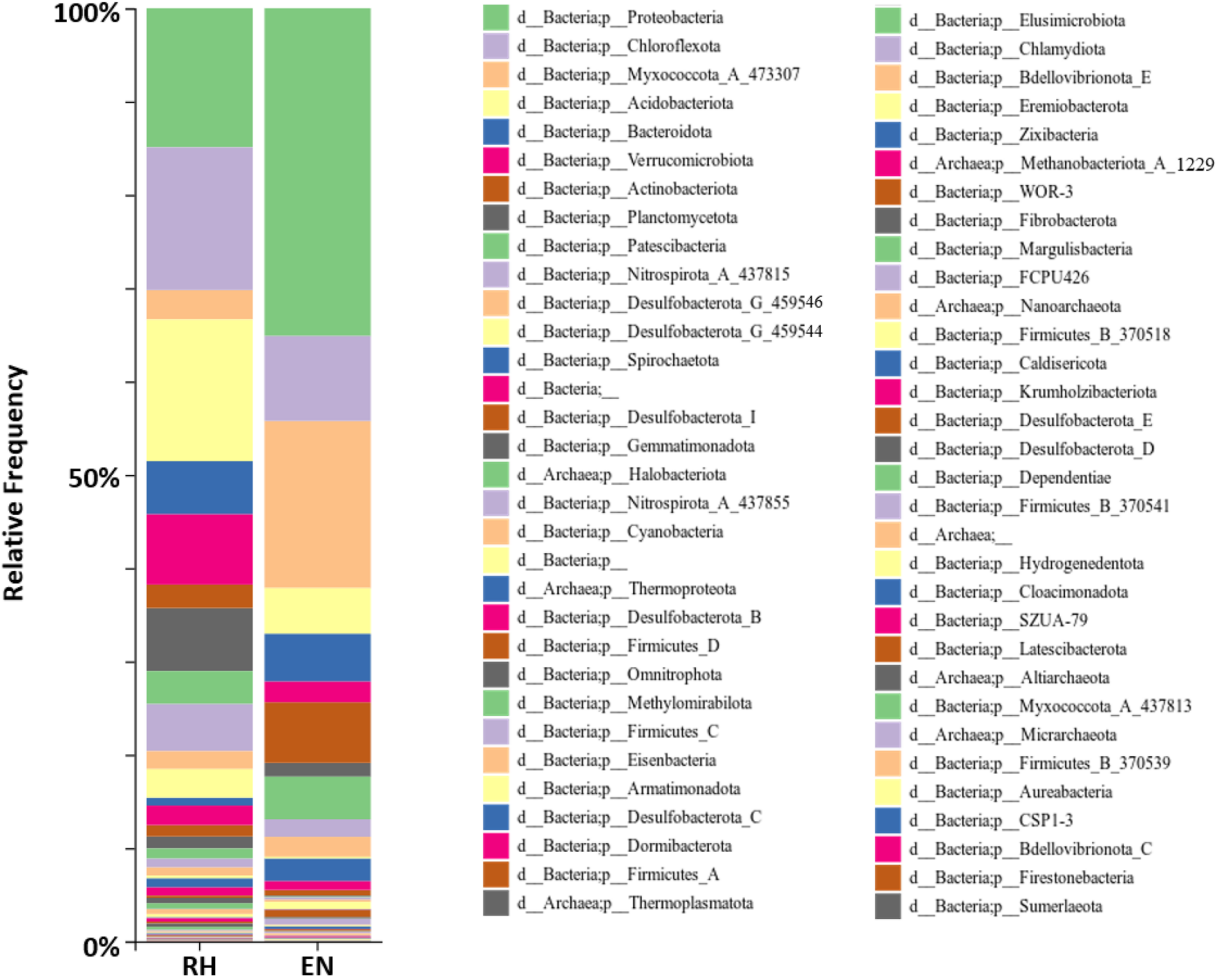
Taxa barplot presenting the relative abundance of bacteria phyla found in rhizosphere (RH) and endosphere (EN) of *indica* root samples.

The distribution of bacteria phyla found in the rhizosphere and endosphere of *indica* samples suggested that most of the bacteria communities found in the rhizosphere and endosphere of rice root were from phylum Proteobacteria, Chloroflexota, Myxococcota, Acidobacteria, Bacteroidota, Verrucomicrobia, Actinobacteria and Planctomycetota (Fig. 2).

Furthermore, the relative abundance of several bacteria phyla was found to be significantly higher in the endosphere than in the rhizosphere including the Proteobacteria, Myxococcota, Spirochaetota, Actinobacteria and Firmicutes via the ANCOM differential abundance test (Supplementary Figure 1, Supplementary Data S5). In contrast, the relative abundance of bacteria from phyla Verrucomirobia, Acidobacteria, Desulfobacterota, and archaea from phyla Thermoproteota were found to be higher in the rhizosphere compared to the endosphere.

Proteobacteria, Chloroflexota and Acidobacteria were the most abundant phyla in both the rhizosphere and the endosphere (Fig. 2). Another microbiome study in rice roots also observed a similar trend in those three phyla^15^. Those phyla were also found to be the most abundant phyla in two pioneer plants, *Andropogon glomeratus* and *Cheilanthes aemula*, found inside and outside of El Chichón volcano in Mexico^34^. This might suggest that those phyla are very important taxa that are shared among many terrestrial ecosystems.

Proteobacteria was the most abundant phyla found in the soil and is highly diverse^35^. Most Proteobacteria that are found in both rhizosphere and endosphere of cultivated and weedy rice were from the order of Rhizobiales and Burkholderiales (Supplementary Data S2 and S3). Members of the order Rhizobiales are beneficial in oxidizing nitrogen and methane, helping in nodulation in legume plants and producing phytohormones and precursors metabolites^36–39^. Those capacities help to support plants with absorbable nutrients and help to maintain nutrient cycles in the field, while members of Burkholderiales are well known for their plant growth promoting effect^40^. Acidobacteria is widely spread across various terrestrial environments including grassland, tundra, agricultural, peatland, desert and forest soils^41^. Acidobacteria helps cycling organic matter in the field including carbon, nitrogen, phosphate, sulphur and in the degradation or decomposition of plant residues including cellulose, polysaccharides and organic acids^42^. Those processes are significantly important in promoting plant growth and supporting healthy soil. Similarly, Chloroflexota, previously known as Chloroflexi, is a metabolically diverse bacterial group that plays important roles in nitrogen, phosphorus and carbon cycling^43^. Chloroflexota is associated with a wide range of natural environments and found in even those with such extreme conditions as hot springs, tundra, hypersaline mats and lake sediments^44^. Therefore, Proteobacteria, Chloroflexota and Acidobacteria appeared to be integral communities in the terrestrial ecosystem.

### Beta diversity analysis

Beta diversity analysis was performed to investigate the difference between sample groups. In this study, the Aitchison distance matrix was used to carry out the beta diversity analysis and included the high level of sparsity which reflects the nature of microbiome data set. The Adonis test which is multifactorial PERMANOVA was performed on the group of samples considering the effect of field and root compartment, as well as the effect of field and rice type.

**Figure 3:**
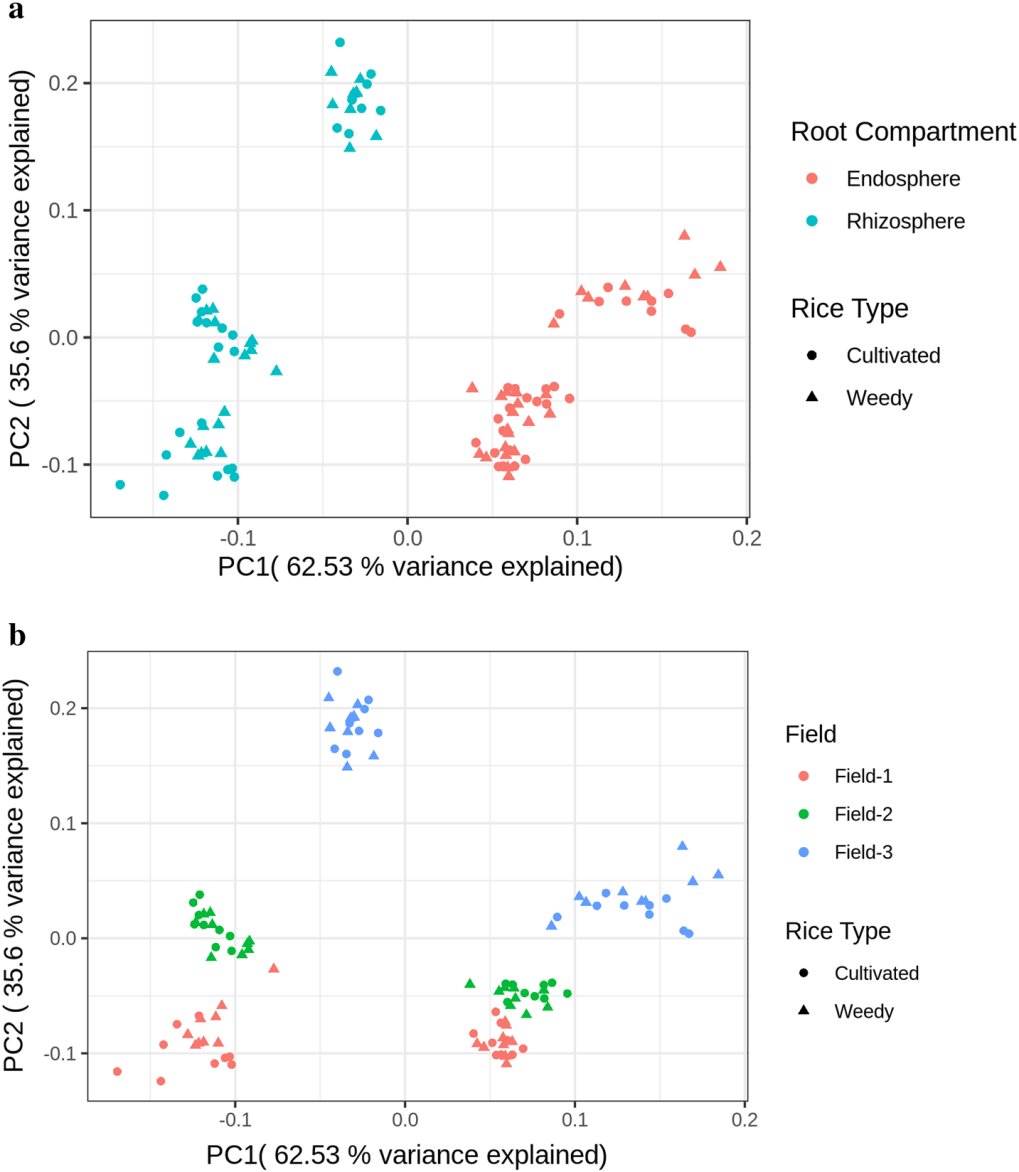
(**a**) PCoA plot using Aitchison distance of cultivated and weedy rice coloured by root compartments. (**b**) PCoA plot using Aitchison distance of cultivated and weedy rice coloured by fields.

The microbial communities vary significantly between different fields and different root compartments (Supplementary Table S1), with the root compartmentalization factor (46.30%, *p* = 0.001) contributing slightly more to the diversity than the field factor (43.62%, *p* = 0.001). Consistent with the Adonis test, the PCoA plot showed a clear separation between rhizosphere and endosphere (Fig. 3a). This again, echoes previous findings that root compartment plays an important role in microbial variation^15,16^. A distinct separation was also observed among field 1, field 2 and field 3 (Fig. 3b), in accordance with a previous study in Ghana presenting variation of microbes across different geographical locations^17^. The geographical locations can be further related to the climate, rainfall, environmental conditions, and even the common agricultural practice adopted in those regions. Hence, different fields in different and/or same geographical areas can show variability in their microbial diversity.

On the other hand, the microbial communities do not vary significantly between the rice types (0.06%, *p* = 0.902 in Supplementary Table S2), which is also observed in the PCoA plot showing no distinct clustering between cultivated and weedy rice (Fig. 3a and b). Interestingly, a greenhouse experiment comparing the rhizosphere of cultivated and wild rice grown in the pot found that the rhizosphere of cultivated rice was decreased in diversity and enriched with certain microbes indicating selection process throughout the domestication^18^. However, we did not detect such a difference in the microbial composition between weedy and cultivated rice that were examined here. Weedy rice is known to closely mimic cultivars in terms of biological and physiological characteristics in order to survive and propagate in rice fields. Furthermore, the weedy rice in this study was sampled from the same field as the cultivated rice, so the weedy rice most likely originated from the cultivars in the field, giving rise to the possible explanation for high similarity of root microbiomes between the weedy and cultivated rice.

### Differential abundance testing based on the predictive functional profile from 16S amplicon data

Studies suggested that invasive plants can alter nutrients and elemental cycling in the soil and selectively recruit certain microbes to initiate feedback that is potentially helpful in further invasion^45^. For example, enrichment of *Clostridium* was observed in the endosphere of invasive plant, *Ageratina adenophora* and this microbial assembly might cater advantages for this invasive plant to compete against native plants^46^. A similar strategy but with a different bacteria group was observed in another invasive tree species, *Acacia dealbata*, which recruited more *Bradyrhizobium* to its rhizosphere to increase competitive ability in the invaded habitat^47^. However, ANCOM differential abundance test showed no taxa that is significantly different in abundance in the endosphere of weedy rice compared to that of cultivated rice (Supplementary Figure S2). This suggests that the invasiveness of weedy rice in the field could be contributed by different factors other than the microbial composition of the rhizosphere and endosphere.

In Supplementary Figure S3, the ANCOM volcano plot presents the differential abundance of metabolic pathways obtained from the predictive functional profiling form PICRUSt2. The metabolic pathway possessed by bacteria in the rhizosphere mainly relates to the synthesis of essential metabolites such as aromatic amino acids, ubiquinone, siderophores and the degradation of organic compounds (Supplementary Data S6). Those processes are beneficial to transform nutrients in the soil into forms that can be absorbed and could be used for the growth of the plant and to maintain a healthy environment for the plant. Also, more pathways related to chemical compound degradation and methanogenesis were found in the rhizosphere than in the endosphere (Supplementary Data S1). Methanogenic archaea were more abundant in the rhizosphere than endosphere perhaps because such archaea function under anaerobic conditions. An oxic zone is formed surrounding the roots since oxygen was released by the rice root aerenchyma, while anoxic zone is observed in surrounding bulk soil^48^. An oxic-anoxic region is formed in the rhizosphere which allows aerobic, anaerobic and/or facultative anaerobic microbes to grow.

On the other hand, the metabolic pathway possessed by the bacteria in the endosphere is mainly related to the degradation, utilization and assimilation of various organic compounds such as secondary metabolites and other chemical compounds absorbed by the root (Supplementary Data S6). The degradation of these compounds can lead to the release of energy and can even be useful for plants to cope better with certain stress conditions such as high salinity or metal contamination in the soil^49^. Therefore, the bacteria in each root compartment may differ to serve various purposes to support overall plant growth.

### Microbial diversity in *japonica* rice

**Figure 4:**
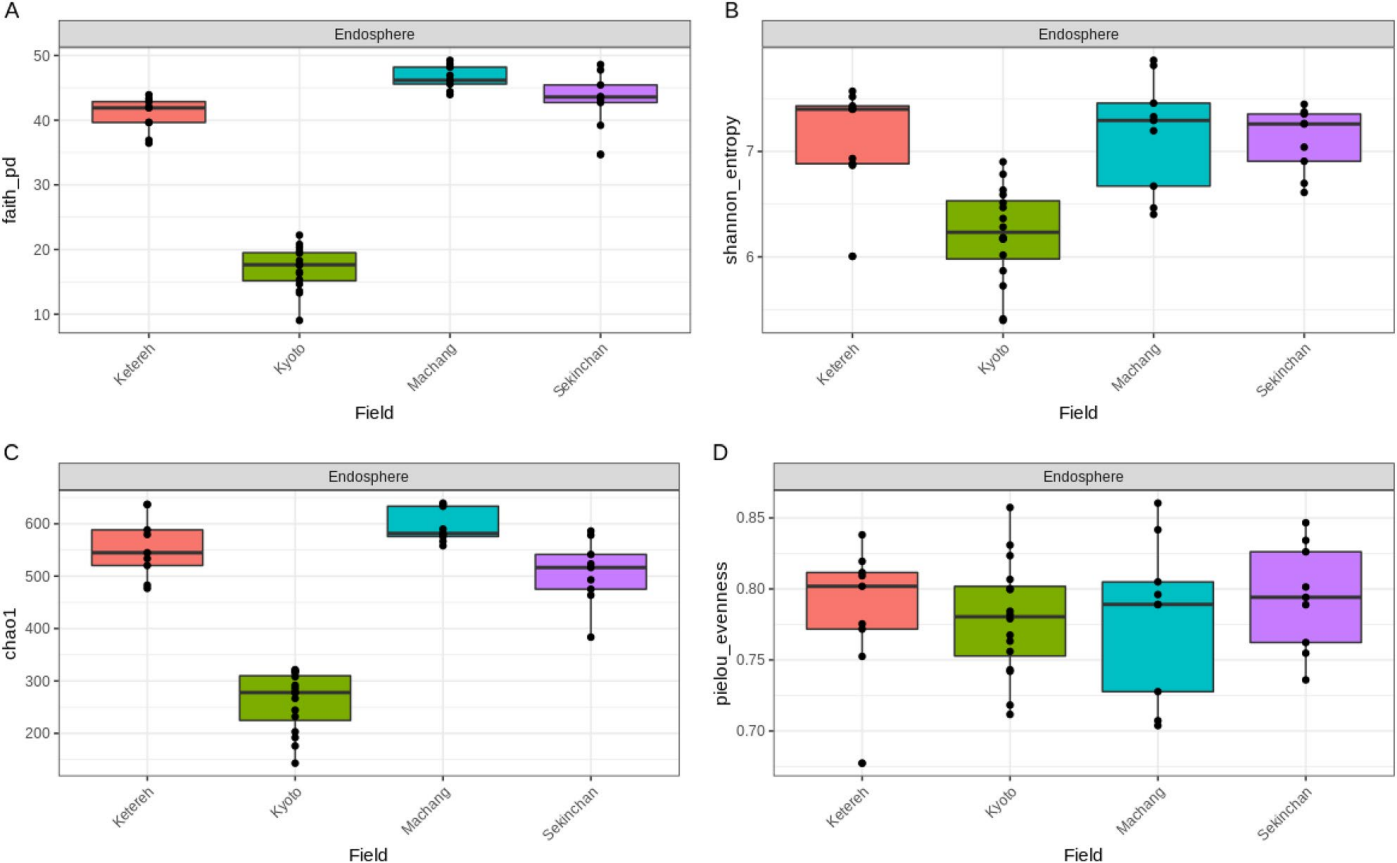
(**a**) Faith’s phylogenetic diversity, (**b**) Shannon diversity, (**c**) Chao1 index indicating community richness and (**d**) Pielou’s evenness index indicating community evenness in the endosphere of *japonica* and *indica* rice.

**Figure 5:**
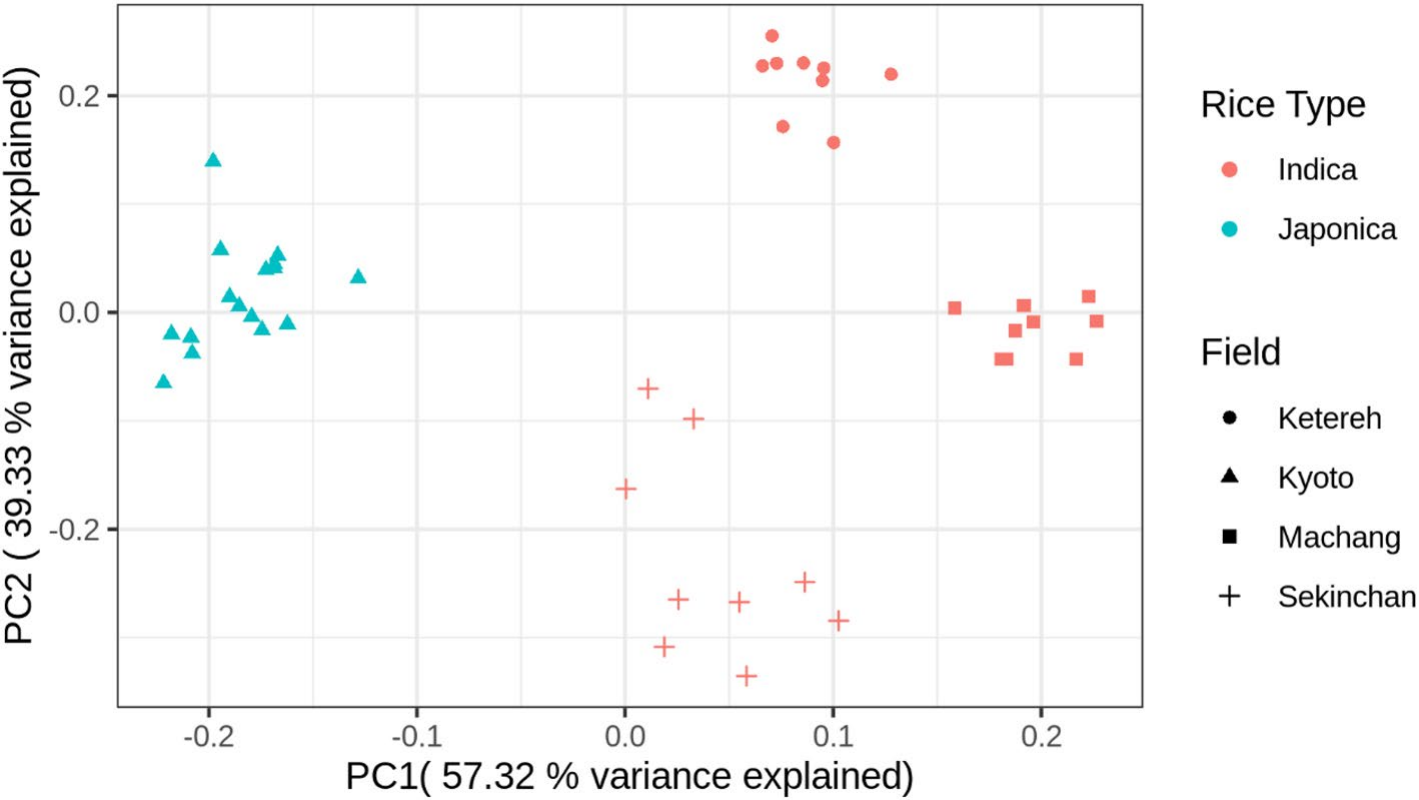
PCoA plot using Aitchison distance for microbial taxa in the endosphere of *japonica* and *indica* rice samples.

Cultivated *japonica* rice grown in Japan was also analyzed for the microbes present in the endosphere. The alpha diversity analysis showed that the microbial richness in the endosphere of *indica* samples was higher than *japonica* samples*,* while the evenness was not significantly different (Fig. 4). Furthermore, the beta diversity using Aitchison distance was used to plot the PCoA suggesting that the *japonica* and *indica* sample groups were significantly different (p = 0.001) forming two distinct clusters (Fig. 5). Although environments could account for some of the variation observed, the microbial difference between *japonica* and *indica* might also suggest the genotype effect, in which host genetics could lead to different metabolic processes that favour a particular subset of microbes to be retained and pass through over a long domestication period of rice. These observations echoed the study by Zhang et al.^50^ that found a separation between *indica* and *japonica* grown in the same field and the results were consistent for two different fields. Also, the alpha diversity was higher in *indica*^50^.

**Figure 6:**
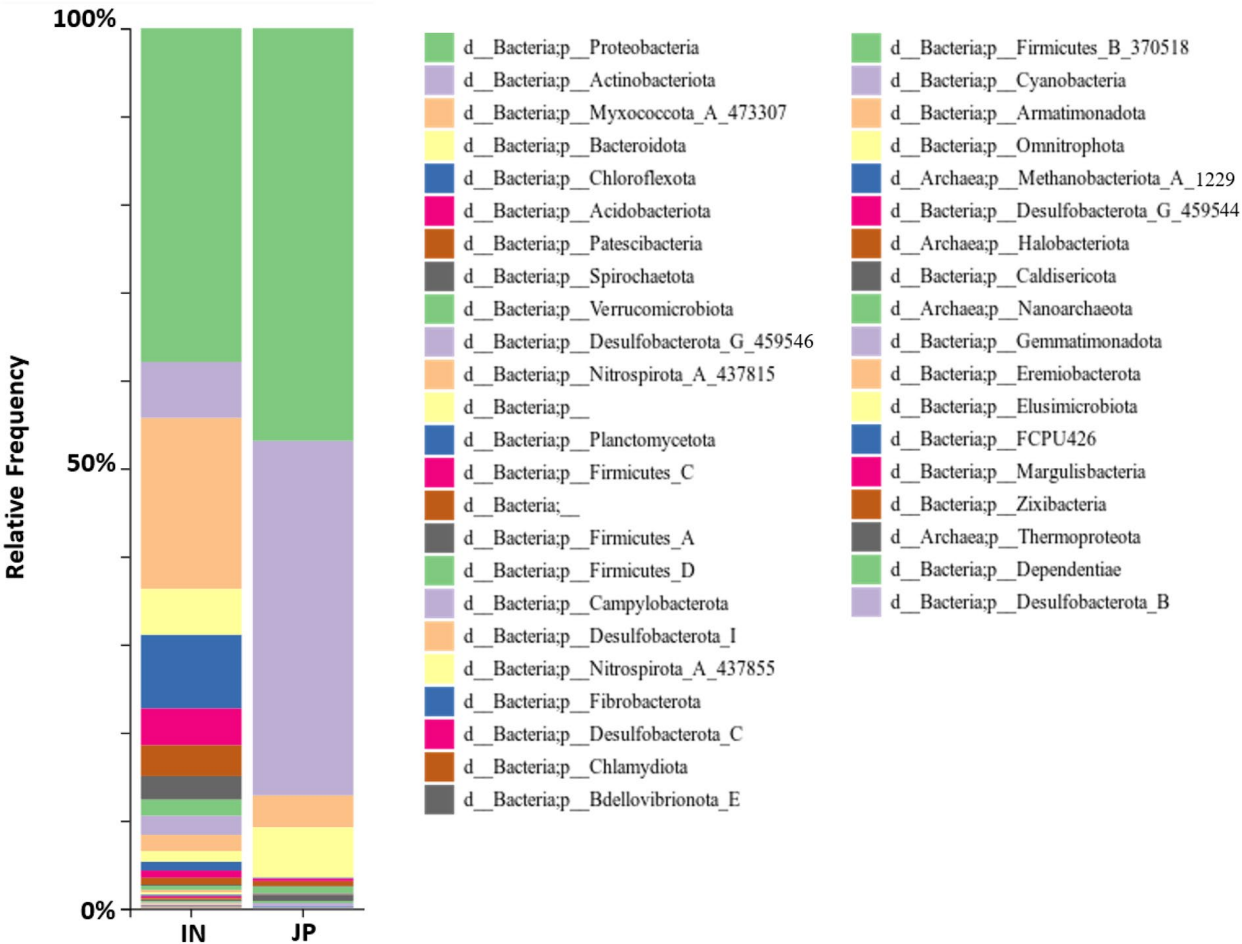
Taxa barplot presenting the relative abundance of bacteria phyla found in the endosphere of *indica* (IN) and *japonica* (JP) rice root samples.

The taxa barplot of bacterial phyla in both *japonica* and *indica* further demonstrates that there was higher diversity in the microbial community in the endosphere of *indica* sample group (Fig. 6). Also, ANCOM volcano plot showed many phyla were found to be differentially abundant in *japonica* and *indica* rice groups (Supplementary Figure S5, Supplementary Data S8). This observation supports the latitudinal diversity gradient (LDG) concept which indicates the increase of species richness from the poles to the equator and this occurs in nearly all organisms including plants, animals and even microorganisms^51^. There are many hypotheses trying to explain this LDG pattern, one of which is related to the higher temperature in the equator or tropical regions which lead to higher rate of metabolism and turnover rate of organic matter that can support ecological dynamics and help to maintain high biodiversity^51^. In this case, *indica* rice subspecies has been well adapted to tropical climates throughout the long domestication process, so much of the bacterial diversity in *indica* could be due to the long acclimatization process in the tropical climate. Despite the differences in bacterial diversity between *indica* and *japonica* lines grown in different environments, a core microbiome for the endosphere could be detected, suggesting these bacteria taxa are important for the rice life-cycle.

### Core microbiome from *indica* rice and *japonica* rice

There are respectively 112 and 42 core microbes (Supplementary Figure S4, Supplementary Data S4) that are shared among the rhizosphere and endosphere of the three fields in Malaysia which might indicate the importance of these microbial groups to support general plant health and growth. Furthermore, there are some microbes that are uniquely found in each field which might be correlated with the environmental factors and agricultural practices in each field. Comparison with the *japonica* results allowed us to further investigate whether there is a core microbiome in the endosphere of rice, despite the genetic difference between both rice varieties.

**Figure 7:**
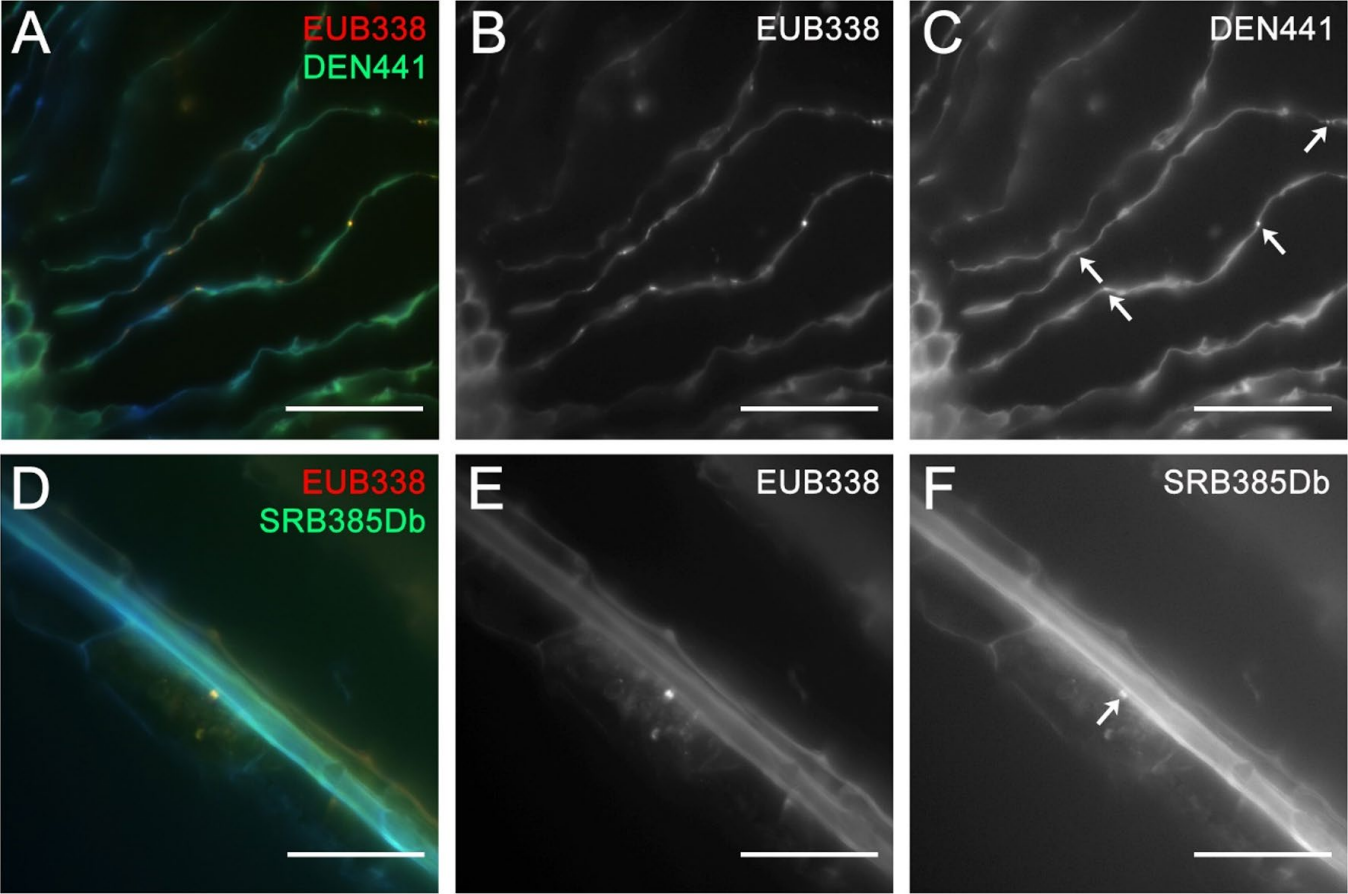
Fluorescence in situ hybridization detecting Rhodocyclaceae – DEN441 and EUB338 signals are colocalized in intercellular space of root aerenchyma of Japanese rice; and Myxococcales – SRB385Db and EUB338 signals are colocalized in intercellular space near the epidermal tissue of Japanese rice.

Despite various differences, there are some microbial groups that were found in all endosphere of both *japonica* and *indica* rice samples, including phylum Myxococcota, family Rhodocyclaceae and order Rhizobiales (Supplementary Data S7). The presence of family Rhodocyclaceae and member of phylum Myxococcota, order Myxococcales were detected in the intercellular space of aerenchyma and epidermal tissue of the *japonica* rice by fluorescence in situ hybridization (FISH) (Fig. 7). This observation of bacteria retained in the plant tissue in samples of both *indica* and *japonica* could be related to specific functions catered by the microbes in relation to plant health and growth. Rhodocyclaceae is well known for the denitrifying property and ability to degrade various organic compounds which are correlated to support plant growth and health in the field. The Rhodocyclaceae is even considered as a suitable candidate to help with wastewater treatment, bioremediation of polluted environments^52^. The Rhodocyclaceae detected may have some role in enhancing the survivability of plants under adverse environmental conditions in the field. In addition, the Myxococcales order includes members who can also fix nitrogen and are abundant in paddy fields^53^. Cleary more detailed characterization of the Myxococcales detected is warranted. The Myxococcales and Rhodocyclaceae were also found in the endosphere of *Arabidopsis* root^54^. Rhizobiales has been found to be highly correlated with nitrogen fixing capability which helps plants to transform the atmospheric nitrogen into the form that can be used by plants^37^. Considering the importance, those phyla could be members of core beneficial phyla for most land plants.

Unfortunately, the short read amplicon sequencing has limited resolution in separating a wide range of microbes, so it has limited ability to determine the core microbes up to species level. Given the difficulties in defining a human microbiome^55^, it is not surprising that a rice core microbiome in the endosphere at the species level cannot be easily ascertained. A future study involving long read sequencing and greater number of weedy and cultivated rice samples can be conducted to capture more bacteria taxa that could have been missed by the short read sequencing.

Weedy rice is known as a natural genetic reservoir for rice with immense amount of genetic variation that has been widely studied in searching for the genes related to the high versatility of this weed against various environmental conditions and its high growth property^56^. At the outset, we expected to find consistent differences in the endosphere microbiome between cultivated *indica* and its conspecific weedy rice. No such difference was observed. This demonstrates that alterations in the endosphere microbiome are not associated with the phenotypic differences between cultivated and conspecific weedy rice.

## Conclusion

Microorganisms in the rhizosphere are more diverse as compared to the endosphere in the rice plants. Different environmental conditions and abiotic factors can contribute to the variation in microbial diversity in different fields. Generally, rice roots may have a selection mechanism in recruiting certain microbes to the endosphere. Despite our expectation of difference, it was found that the microbial composition of the conspecific weedy rice endosphere is highly similar to that of cultivated rice. A possible explanation for this observation is because weedy rice possesses high physiological and morphological similarities to cultivated rice, so the selection towards surrounding microbes may be very similar. Future exploration involving metabolomics would be very useful to get information on the putative functional metabolites that may contribute to invasiveness or increase competitive ability in the invasive plant like weedy rice. Long-read shotgun metagenomic sequencing can be undertaken to obtain more comprehensive taxa at the species level. Despite differences in microbial diversity found between *indica* and *japonica* samples, rice plants retain selection of microbes, which are preferentially found in the root. The selection could involve complex interactions between the roots and the microbes, as well as various biotic and abiotic factors encountered throughout the long domestication of rice.

### Supplementary Information

Supplementary Information 1.

Supplementary Information 2.

## Data Availability

The raw sequencing reads for this study were submitted to the National Centre for Biotechnology Information (NCBI) Sequence Read Archive (SRA) under BioProject PRJNA964501, https://www.ncbi.nlm.nih.gov/bioproject/?term=PRJNA964501.
