# Supplementary material for: Comparative analysis of root associated microbes in tropical cultivated and weedy rice (Oryza spp.) and temperate cultivated rice
Source: Sci Rep. 2024 Apr 26;14:9656. doi: 10.1038/s41598-024-60384-0 (PMC11053024; doi:10.1038/s41598-024-60384-0)
Supplement: Supplementary file 2 — Supplementary Information 2. [file 41598_2024_60384_MOESM2_ESM.docx]

**Supplementary**

**Table 1.** Adonis test for field and root compartment

|  | **R^2^** | **p-value** |
| --- | --- | --- |
| **Field** | 0.436238 | 0.001 |
| **Root Compartment** | 0.463042 | 0.001 |

**Table 2.** Adonis test for field and rice type

|  | **R^2^** | **p-value** |
| --- | --- | --- |
| **Field** | 0.436238 | 0.001 |
| **Rice Type** | 0.000634 | 0.902 |


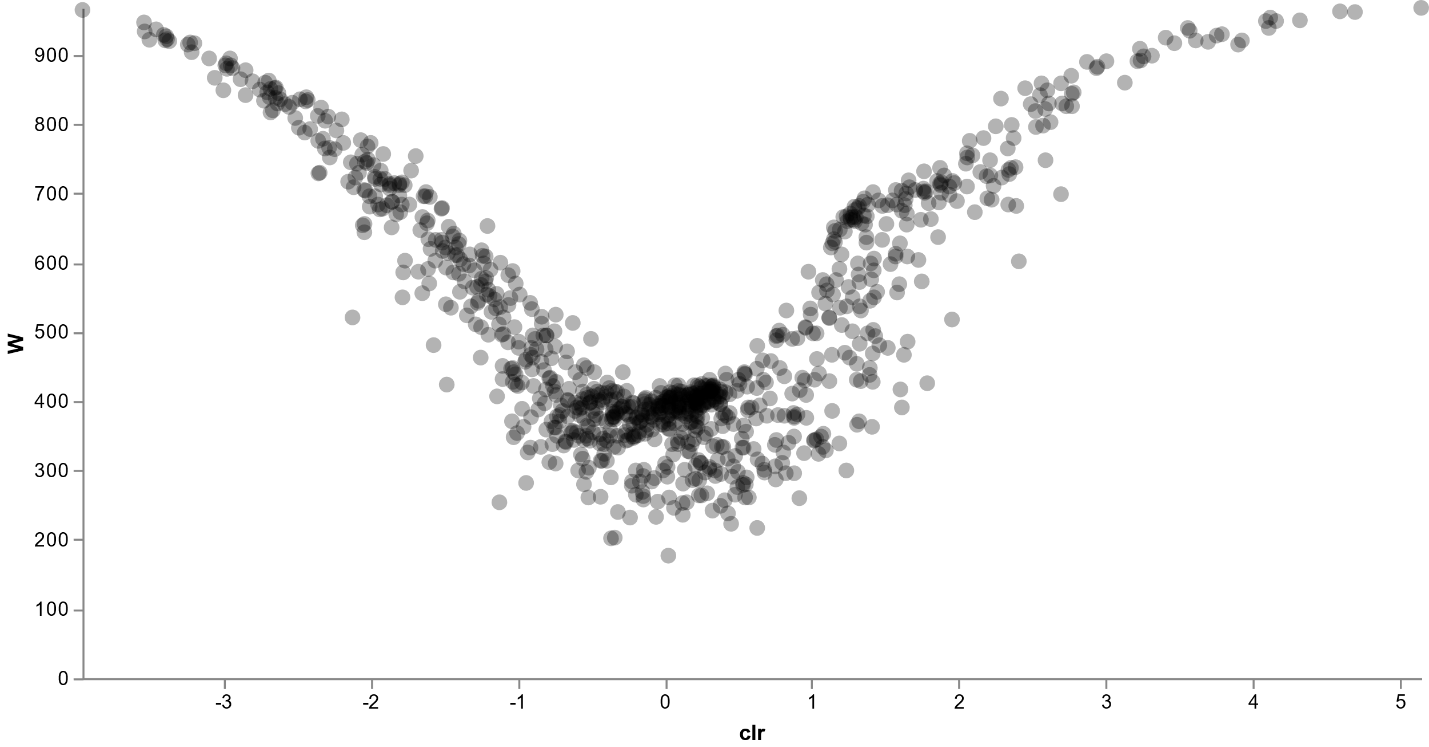


# Figure 1. Ancom volcano plot of differential abundance in bacterial taxa found in the rhizosphere and endosphere of cultivated and weedy rice. W is the ANCOM test statistic and indicates the number of times the null hypothesis is rejected by the analysis. The higher W, the more likely a taxon differs statistically. The clr indicates the effect size change between the compared groups. Statistically significant features as identified by ANCOM are above the blue line. List of taxa are in Supplementary Data S4.


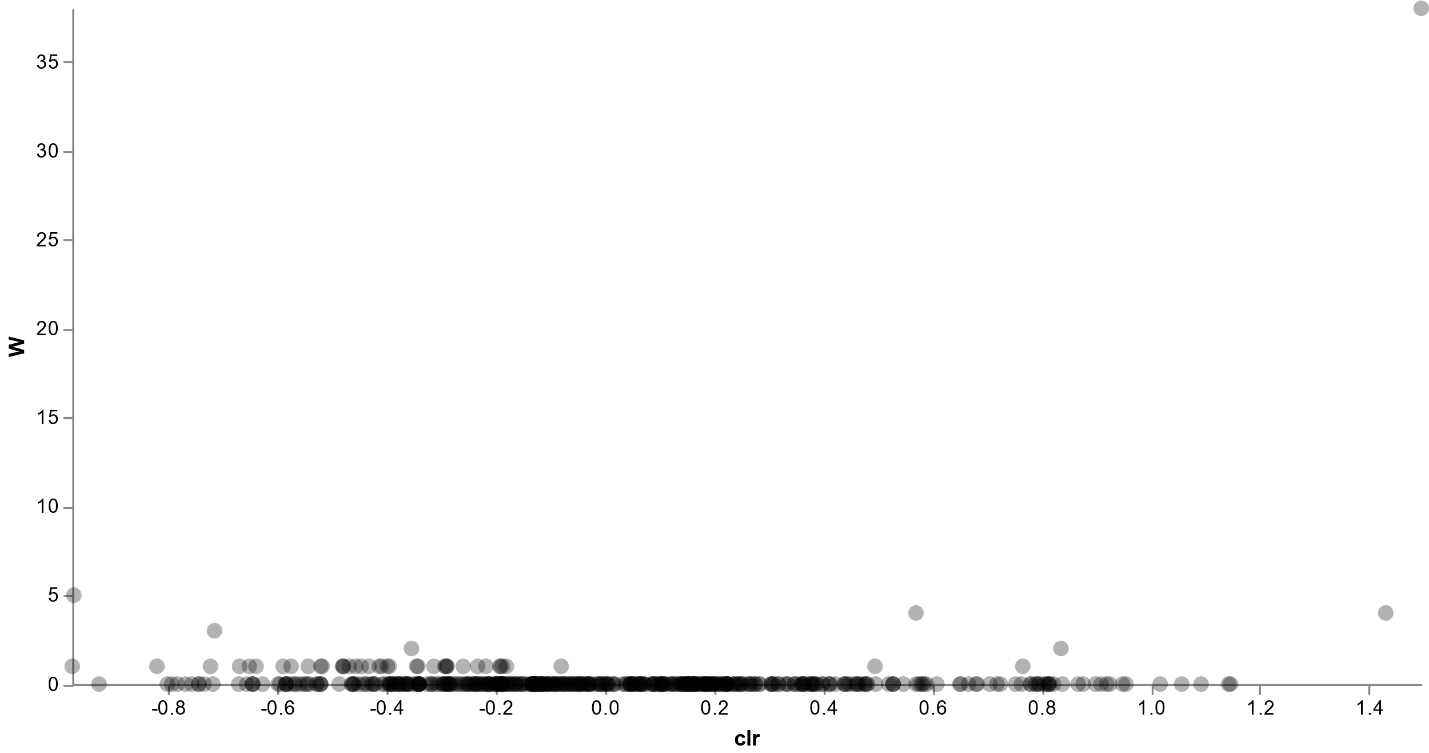


**O_Pedosphaerales**

# Figure 2. ANCOM volcano plot of differential abundance of bacteria taxa in the endosphere of cultivated rice and weedy rice. W is the ANCOM test statistic and indicates the number of times the null hypothesis is rejected by the analysis. The higher W, the more likely a taxon differs statistically. The clr indicates the effect size change between the compared groups. ANCOM statistics detects no significant taxa here.


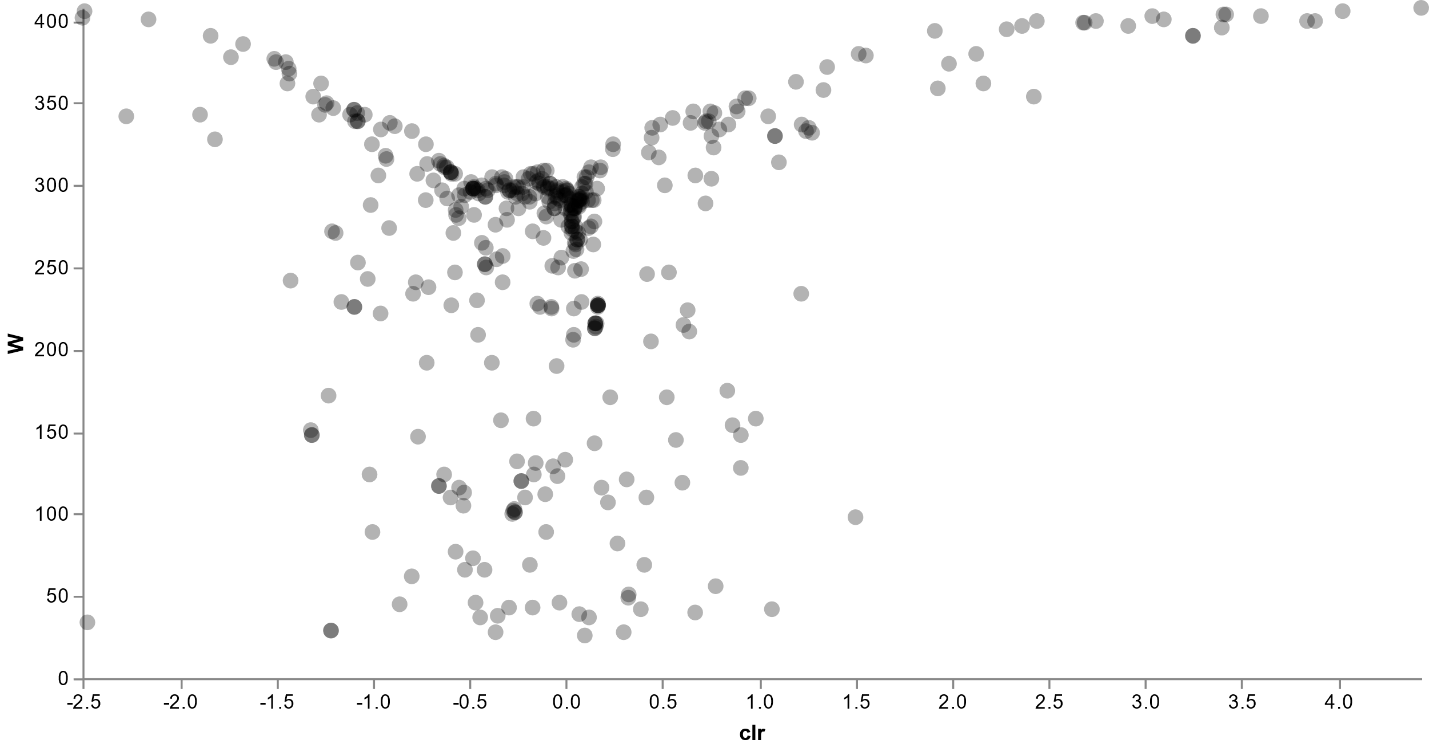


**Figure 3**. Ancom volcano plot of differential abundance in metabolic pathway in the rhizosphere and endosphere. W is the ANCOM test statistic and indicates the number of times the null hypothesis is rejected by the analysis. The higher W, the more likely a pathway differs statistically. The clr indicates the effect size change between the compared groups. Statistically significant pathways as identified by ANCOM are above the blue line. List of pathways are in Supplementary Data S6.


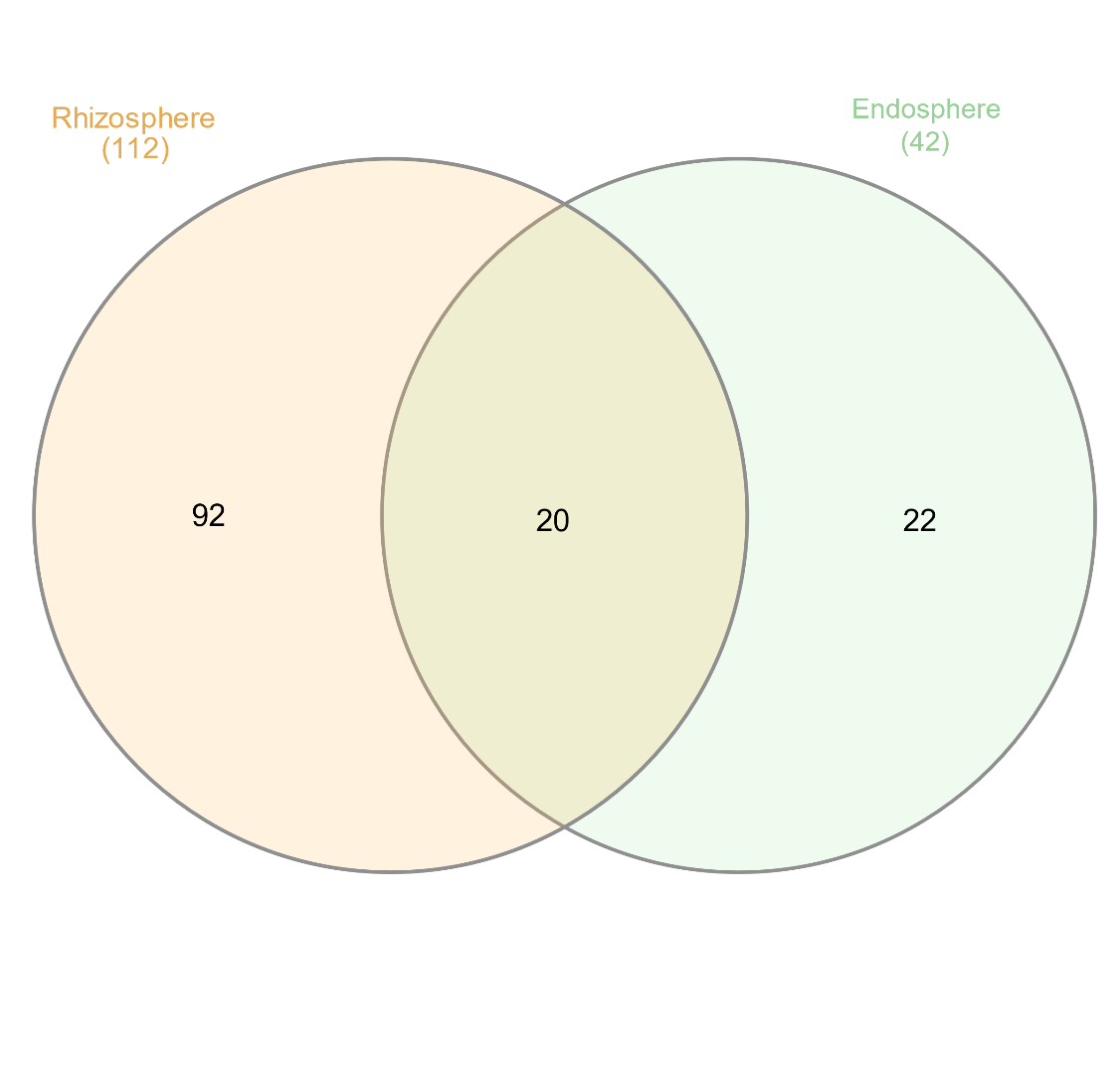


**Figure 4**. Venn diagram of core microbiome found in Malaysia’s sample group in the rhizosphere and endosphere (Supplementary Data S4).


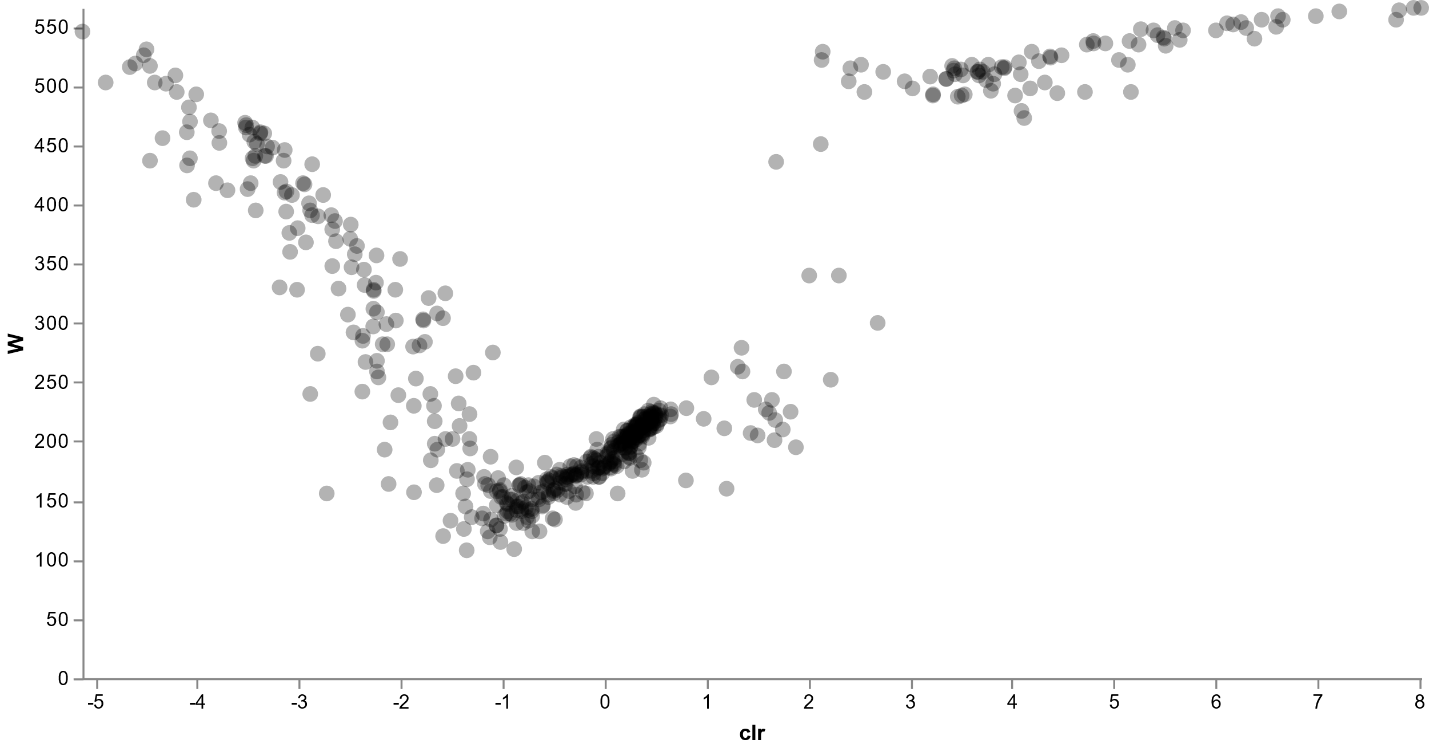


**Figure 5**. Ancom volcano plot of endosphere bacterial community in *japonica* and *indica* rice group. W is the ANCOM test statistic and indicates the number of times the null hypothesis is rejected by the analysis. The higher W, the more likely a taxon differs statistically. The clr indicates the effect size change between the compared groups. Statistically significant features as identified by ANCOM are above the blue line. List of taxa are in Supplementary Data S8.


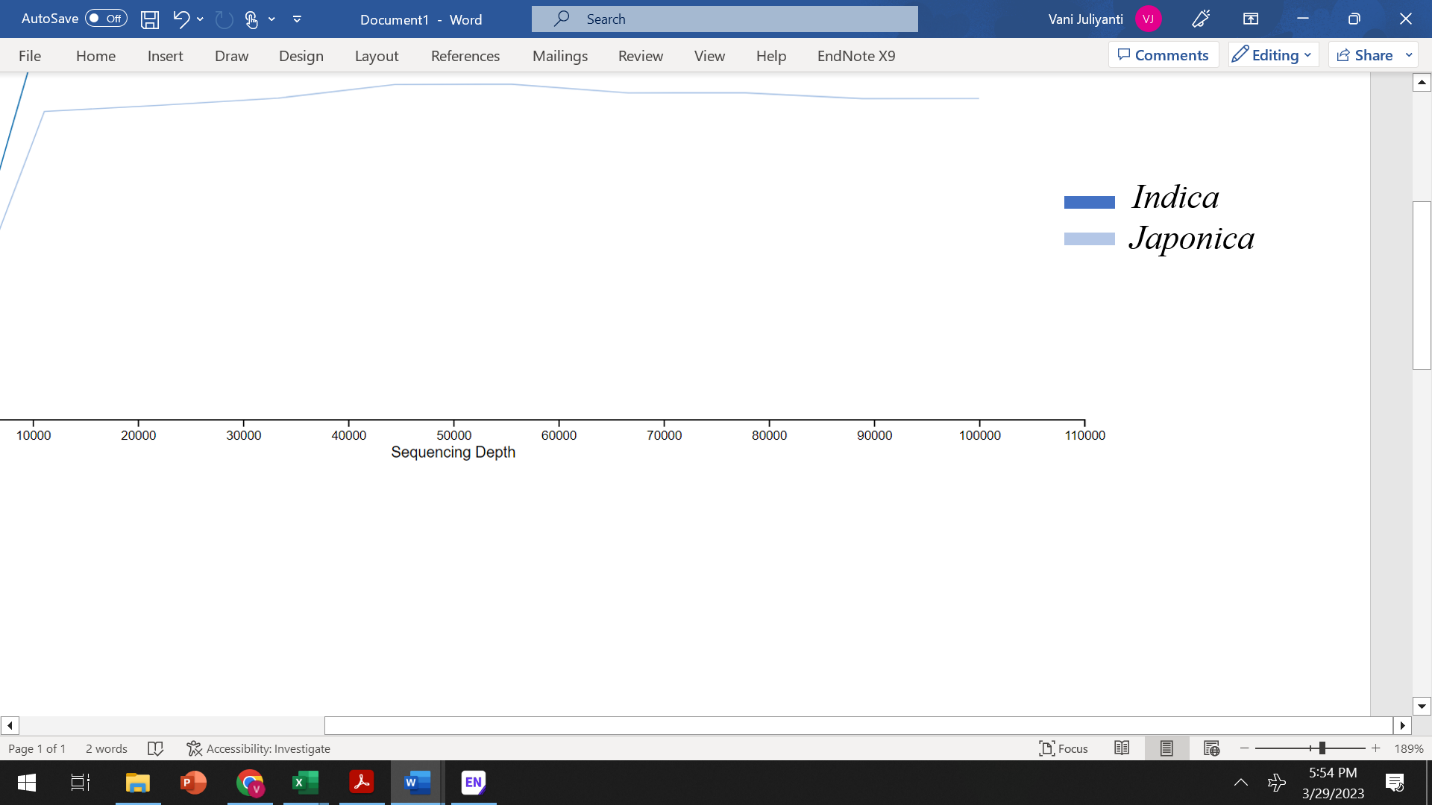

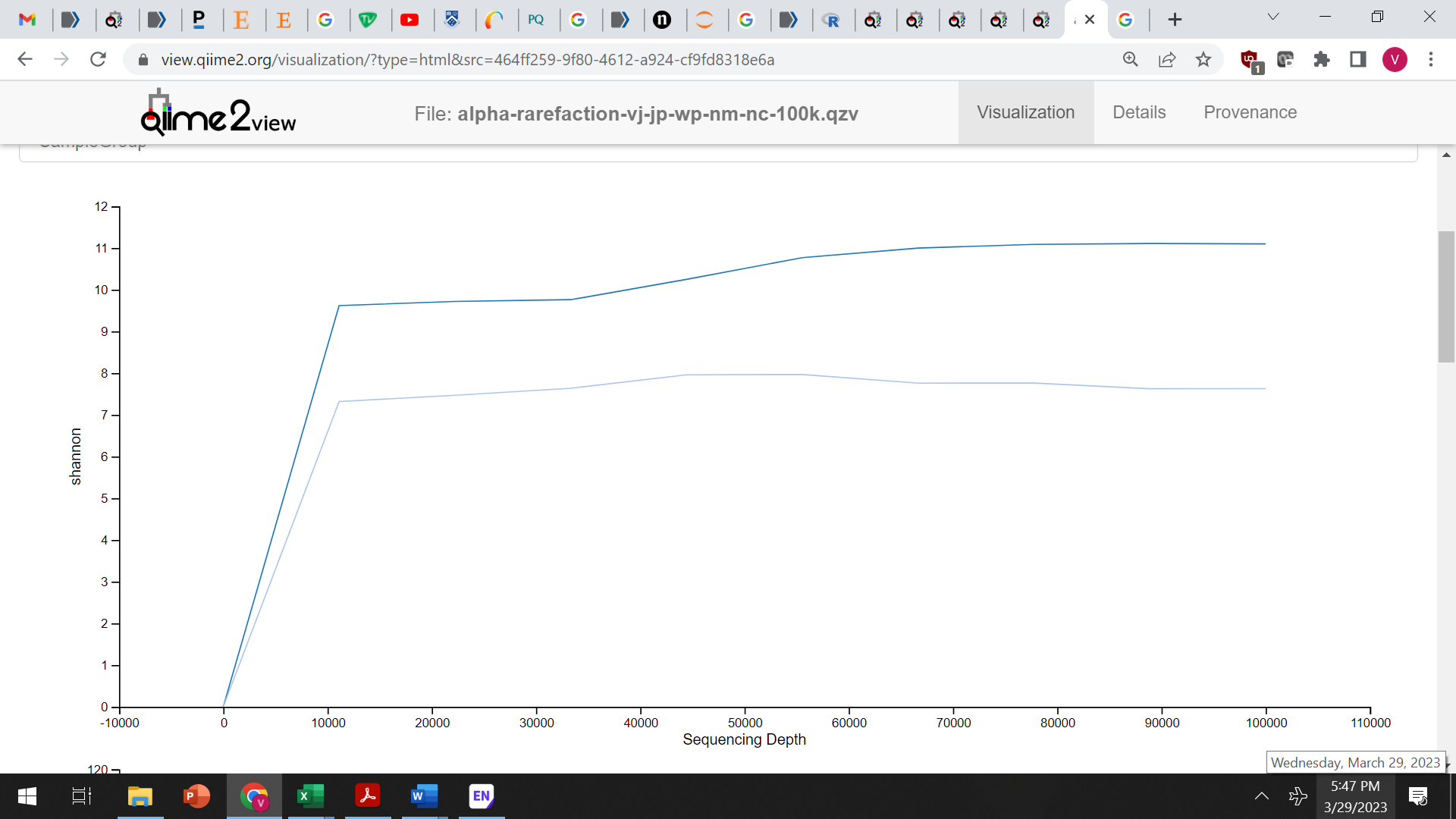


**Figure 6.** Alpha rarefaction plot demonstrating microbial diversity in *indica* and *japonica* sample groups using Shannon diversity index.


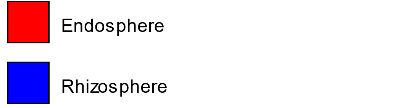

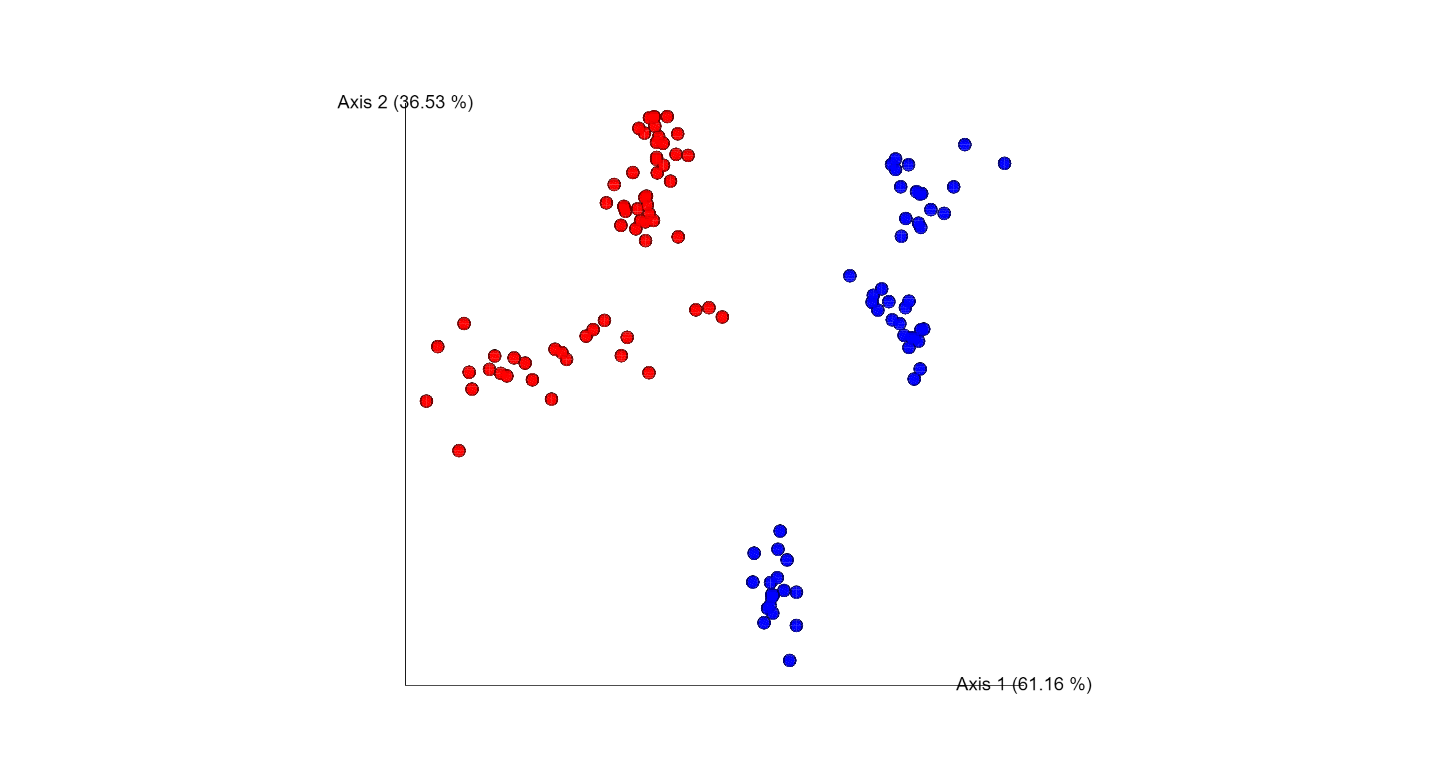


**Figure 7.** PCoA plot using Aitchison distance of cultivated and weedy rice of *indica* samples tested with method used for *japonica* samples
